# FMI and 2D seismic integration for fractured basement reservoir assessment, Geisum area, Gulf of Suez

**DOI:** 10.1038/s41598-025-08278-7

**Published:** 2025-07-05

**Authors:** Yousef Hendawy, Hassan H. Elkadi, Taher Mostafa

**Affiliations:** 1 Faculty of Science, Geology Department, Al-Azhar University, P.O. Box 11884, Nasr City, Cairo, Egypt; 2 Faculty of Science, Geology Department, Al-Azhar University, P.O. Box 11884, Nasr City, Cairo, Egypt

**Keywords:** Unconventional reservoir, Fractured basement, Seismic data, FMI data, Geisum area, Gulf of Suez, Geology, Geophysics

## Abstract

Fractured basement reservoirs represent critical contributors to global hydrocarbon production, with lithologically heterogeneous systems such as weathered granites serving as economically viable targets. In the tectonically active Gulf of Suez rift basin, fractured basement units are increasingly recognized as high-potential reservoirs for hydrocarbon exploration. This study investigates the Geisum Oil Field, a prolific southern Gulf of Suez hydrocarbon province, where basement-hosted production challenges conventional reservoir paradigms. A multidisciplinary approach combining advanced geophysical well logs (including Formation MicroImager [FMI] and resistivity anisotropy analysis) with 2D seismic interpretation was employed to (1) delineate conductive fracture networks, (2) quantify fracture aperture distributions, and (3) resolve structural controls on reservoir heterogeneity. Results identify three dominant fracture orientations—NE–SW, NW–SE, and ENE–WSW—aligned with regional stress regimes. Quantitative analysis reveals a maximum fracture aperture of ~ 0.7 mm within the uppermost basement interval, correlating with enhanced porosity (φ) and permeability (k) zones. Fault intersection geometries were found to amplify fracture density, creating interconnected conduits that optimize reservoir quality. However, kinematic analysis of fault systems highlights potential compartmentalization risks, as insufficient fault seal integrity may permit hydrocarbon migration along reactivated fault planes. These findings underscore the dual role of tectonic fracturing in basement reservoirs: while fracture networks enhance storage and flow capacity, dynamic fault systems necessitate rigorous seal evaluation to mitigate leakage hazards. This work provides a framework for de-risking basement reservoir exploration in rift-related settings globally.

## Introduction

Conventional reservoir assessment, encompassing porosity and water saturation estimation, remains a cornerstone of hydrocarbon reserve evaluation^1–6^. However, the global energy landscape is increasingly shifting towards exploiting hydrocarbons from complex unconventional reservoirs, particularly fractured basement plays. These reservoirs present unique challenges and opportunities for geologists and petroleum engineers. Unconventional reservoirs, such as those in Algeria, China, Vietnam, Canada, India, Yemen, and Egypt, are widespread globally. Fractured basement reservoirs are governed primarily by two key factors: (1) the nature and intensity of tectonic movements, and (2) the lithology of the Precambrian basement rocks, which dictates their susceptibility to fracturing and the development of dense fracture networks^7,8^. Brittle crystalline rocks like quartzites and granites are highly conducive to fracturing under tectonic stress, making them favorable reservoir targets, whereas more elastic metamorphic rocks like gneisses and schists are generally less prone to forming extensive fracture networks^9^.

**Fig. 1:**
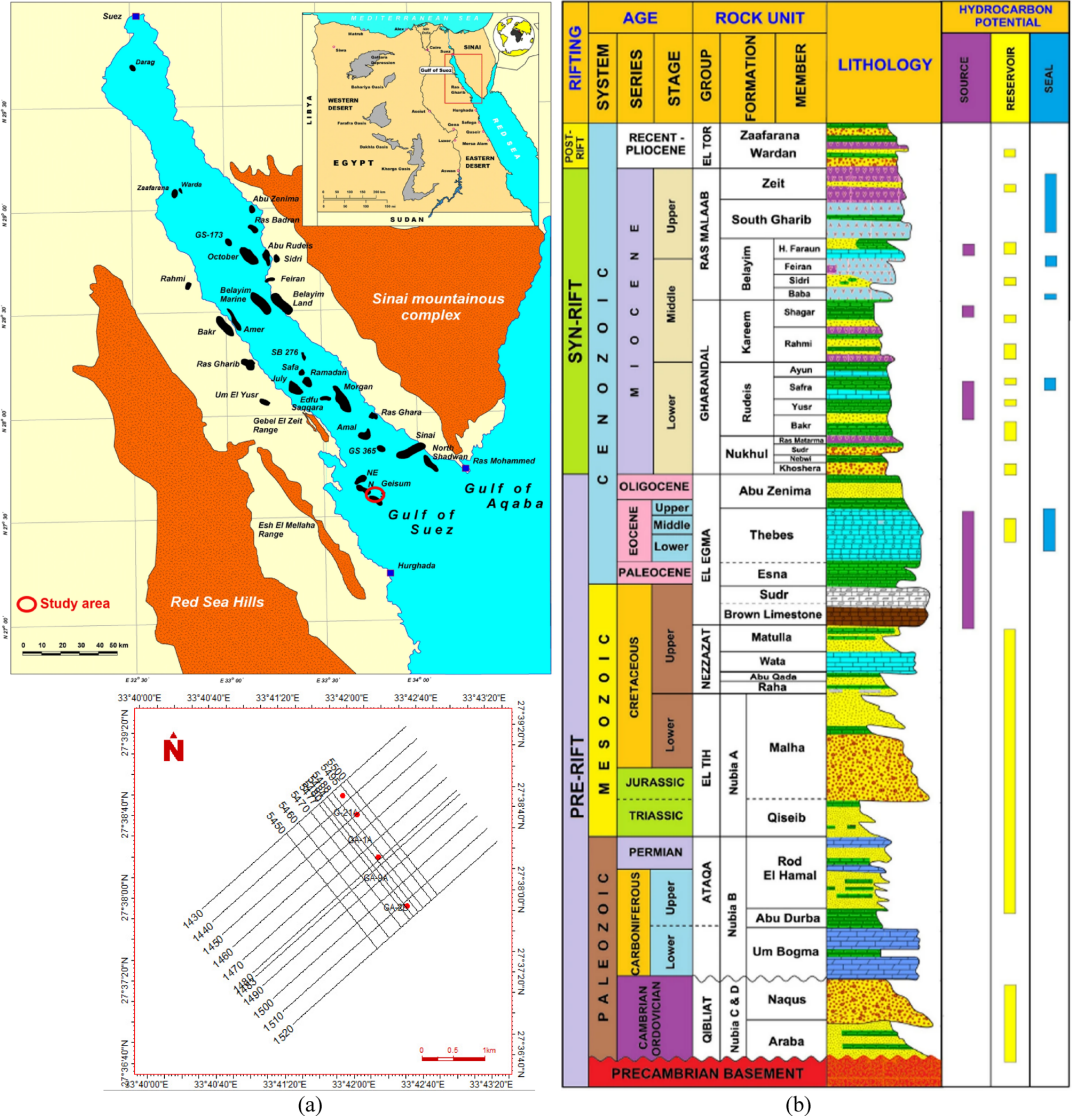
(**a**) Location map of the study area modified after^33^, showing seismic data and well locations. (**b**) Stratigraphic column of the study area modified after^17,18^.

The Gulf of Suez rift basin exemplifies a prolific province for oil production from fractured Precambrian basement reservoirs, with significant contributions from fields such as Zeit Bay and Geisum^10–13^. Motivated by the established production potential of fractured basements in this region, this study focuses on characterizing the fractured basement in the South Geisum oil field (Fig. 1a). The Petrogulf Miser Company began developing the South Geisum oil field in 1988 by drilling four wells and discovering the Precambrian basement reservoir in the northeast of the field, which contains high-relief basement occurrences. Granite and felsic dikes, which intrude granite in the southern Geisum field and are considered excellent reservoirs owing to their brittle rock behavior, primarily compose the basement^10^. The calculated oil reserves from South Geisum were approximately 17 MMSTB and 8 MMSTB from the fractured basement^10,14^. The average daily production from Geisum before the cessation of production was 5500 BOPD, after which it was completed. The production commenced from fields on 28 April 1995, with average daily rates of 15,000 BOPD^14^. Tectonic activity within the Gulf of Suez rift has intensely deformed these basement rocks, creating complex fault and fracture systems across multiple orientations^15–17^. Resolving this subsurface structural complexity—specifically determining fracture/fault orientations and dips, fracture apertures, and fracture density—is critical for effective field development, optimal well placement, wellbore stability, production optimization, and completion design. To address this challenge, the current study integrates available borehole image logs (Formation MicroImager—FMI) and 2D seismic data to qualitatively and quantitatively investigate and evaluate the basement reservoir in the South Geisum field. This study defines four primary objectives: (1) To systematically identify and characterize fractures, faults, and structural discontinuities along the borehole wall utilizing FMI data. (2) To quantify fracture orientation, intensity, and spatial distribution through statistical analysis of FMI data. (3) To characterize sedimentary features (including bedding planes, laminations, and depositional environments) via high-resolution FMI image analysis. (4) To systematically integrate FMI-derived structural and sedimentological interpretations with seismic data analysis for comprehensive subsurface characterization.

### Geologic setting

The Gulf of Suez is a prominent geological formation situated between the Sinai Peninsula and Egypt’s Eastern Desert. It constitutes a segment of the extensive Red Sea Rift system, which continues southward into the Red Sea. The Gulf of Suez is a complicated graben and half-graben system distinguished by normal faults and inclined fault blocks^18^. Three large-scale half-grabens, which alternate in polarity along the rift axis, compose the Gulf of Suez rift, a configuration common to most active and failed continental rifts^19^. Moustafa^20^ first recognized this feature in the Gulf of Suez on the basis of dip meter data and bedding attitudes. Within the southern half-graben, most fault blocks are rotated with dips to the SW, and normal faults with a rift trend are downthrown to the NE^21,22^. The structural pattern reverses in the central half-graben, with most structural dip to the NE, and returns to the SW dip in the northern half-graben. The studied Geisum oilfield lies in Zeit Province (NW-tilted fault blocks and SE-dipping normal faults)^21–25^.

The Gulf of Suez is a dynamic rift basin whose stratigraphic sequence records a long and intricate history of tectonic evolution and sedimentary deposition. Early investigations, coupled with modern seismic and well-log data, reveal a tripartite stratigraphic framework: pre‑rift, syn‑rift, and post‑rift sequences^26,27^. The pre‑rift deposits typically consist of older, relatively undeformed sediments that predate the onset of extension. In contrast, the syn‑rift sequence records dramatic tectonic stretching, faulting, and rapid subsidence, which led to the deposition of clastic, evaporitic, and carbonate units. The post‑rift phase, characterized by a relative stabilization of tectonic forces, shows a gradual transition towards thermal subsidence with sediment accumulation that refines our understanding of basin evolution^15,21,25,28–34^, as shown in Fig. 1b. The thickness, areal distribution, lithology, hydrocarbon importance, and depositional environments of these units vary, e.g.,^35–38^. In the Geisum oil field, the sedimentary sequence ranges in age from Paleozoic to recent^14^. The reservoirs in the Geisum oil field extend from prerift sediment (Nubia sand and basement rock) and Miocene clastic deposits (Rudeis and Nukhul formations). The interesting basement rock is a Precambrian prerift mega-sequence succession. It is composed mainly of alkali granite, quartz diorite, granodiorite, syenogranite, and andesite porphyry dissected by means of dykes, fractures and joints^10^. The geological setting and weathering process performed a significant phase in creating and developing the pre-Cambrian reservoir^10,14,38^.

## Materials and methods

**Fig. 2:**
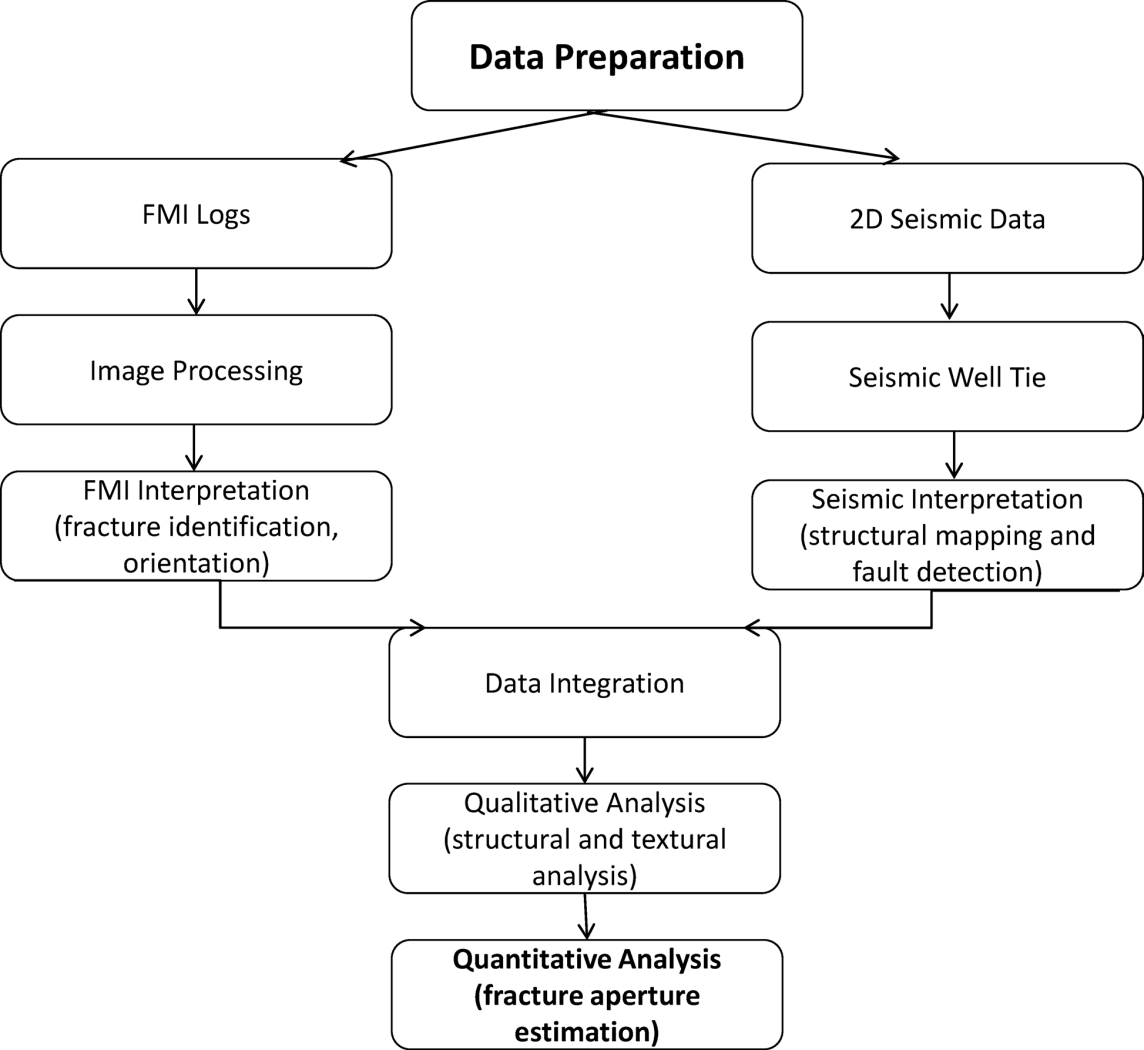
Schematic Representation of the analytical workflow employed in this study.

Four wells and twenty 2D seismic lines were supplied by Petrogulf Misr Petroleum Company and approved by the Egyptian General Petroleum Corporation (EGPC) to accomplish the objectives of the present work (Fig. 1a). The twenty 2D seismic lines are represented by 11 NE‒SW direction sections (inline-seismic) and 9 NW‒SE direction sections (cross-line seismic), as shown in Fig. 1a. Complete set of well log data for the four given wells, in addition to FMI data for two wells (well GA-1A and well G-21A), which include four resistivity pads, four resistivity flaps and their orientations. Seismic interpretation plays a crucial role in the integration of hydrocarbon development strategies within research. This facilitated the identification of hydrocarbon trapping and the delineation of reservoir boundaries. Fault trends can serve as a guide for fracture orientations. The integrated study of seismic interpretation and wellbore imaging is essential for identifying unconventional reservoirs^39,40^. The workflow outlined in Fig. 2 demonstrates a structured integration of FMI and 2D seismic data, optimizing basement reservoir characterization. A thorough analytical evaluation was performed via software tools such as Petrel 2017 (Schlumberger), TechLog 2015 (Schlumberger), and Interactive Petrophysics 2018 (Senergy) programs. The evaluation has focused on identifying geological features (bedding planes, faults, and fractures) and quantifying measurements such as orientation, dip, and fracture aperture.

### Seismic interpretation

The interpretation of typical seismic data is initiated by seismic-to-well ties. The process of tying seismic data to well information is crucial for seismic interpretation and inversion and is frequently neglected yet vital for precise reservoir characterization. The process involves correlating well log data with seismic data to achieve satisfactory alignment between synthetic seismograms and actual seismic data. This correlation establishes a time‒depth relationship, which is essential for constructing accurate subsurface models. Furthermore, seismic coupling assists in identifying offset-dependent wavelets required for seismic inversion, thereby ensuring that the seismic data accurately represent subsurface properties. Strong seismic-to-well ties enable geoscientists to utilize seismic data effectively for subsequent analyses, including seismic inversion and reservoir modeling, thereby facilitating more informed decision-making in exploration and production^40–45^.

In the following step, key horizons or reflectors are identified on the seismic sections either manually or via automatic picking algorithms. Horizon picking, and fault interpretation are essential elements of seismic data interpretation and are vital for comprehending subsurface geology. Horizon picking involves the identification and correlation of continuous seismic reflectors, which are indicative of geological strata or boundaries, across various seismic sections. This method clarifies the structure and stratigraphy of the subsurface, offering significant insights into geological history and possible reservoir zones. Precise horizon selection necessitates meticulous examination of seismic properties, including amplitude, phase, and continuity, to guarantee uniform and dependable interpretations. Fault interpretation involves the identification and delineation of faults, which are discontinuities in seismic data resulting from tectonic activity. Faults can profoundly affect reservoir connectivity and fluid dynamics, rendering their precise detection and characterization crucial for efficient reservoir management. Seismic properties such as coherence, variance, and dip can assist fault detection and elucidate fault geometry and displacement. Together, horizon picking and fault interpretation enhance the development of a comprehensive geological model, which is essential for informed decision-making in hydrocarbon exploration and production. By synthesizing these interpretations with other geological and geophysical data, geoscientists can cultivate a more precise and comprehensive understanding of the subsurface, thereby enhancing exploration and development methods^1,2,30,42^.

Once horizons are picked, they are mapped to create a three-dimensional view of the subsurface geology, which is essential for understanding the distribution of reservoirs and guiding exploration and production activities. Time-based seismic data often undergo depth conversion to provide a more accurate representation of the subsurface^2,42^. Horizon mapping is a fundamental step in seismic interpretation, enabling geoscientists to construct comprehensive geological models and make informed decisions in hydrocarbon exploration and production^2,42,46,47^.

### Image processing

The interpretation of FMI involves numerous critical processes to guarantee precise and dependable outcomes. The raw FMI data are first subjected to preprocessing, which involves noise reduction and calibration to rectify borehole effects and tool artifacts. This phase guarantees that the photos precisely depict the formation. After preprocessing, picture enhancement techniques such as filtering, and contrast correction were implemented to improve the visibility of geological features. The subsequent phase entails feature extraction, during which structural components such as fractures, bedding planes, and faults are recognized and delineated. Advanced algorithms and edge-detection methods are frequently utilized to automate this procedure and enhance precision. The analyzed data are amalgamated with additional geological and geophysical information to construct a comprehensive subsurface model. This integration facilitates the comprehension of reservoir characteristics and dynamics, assisting in exploration and production determination^48,49^.

**Fig. 3:**
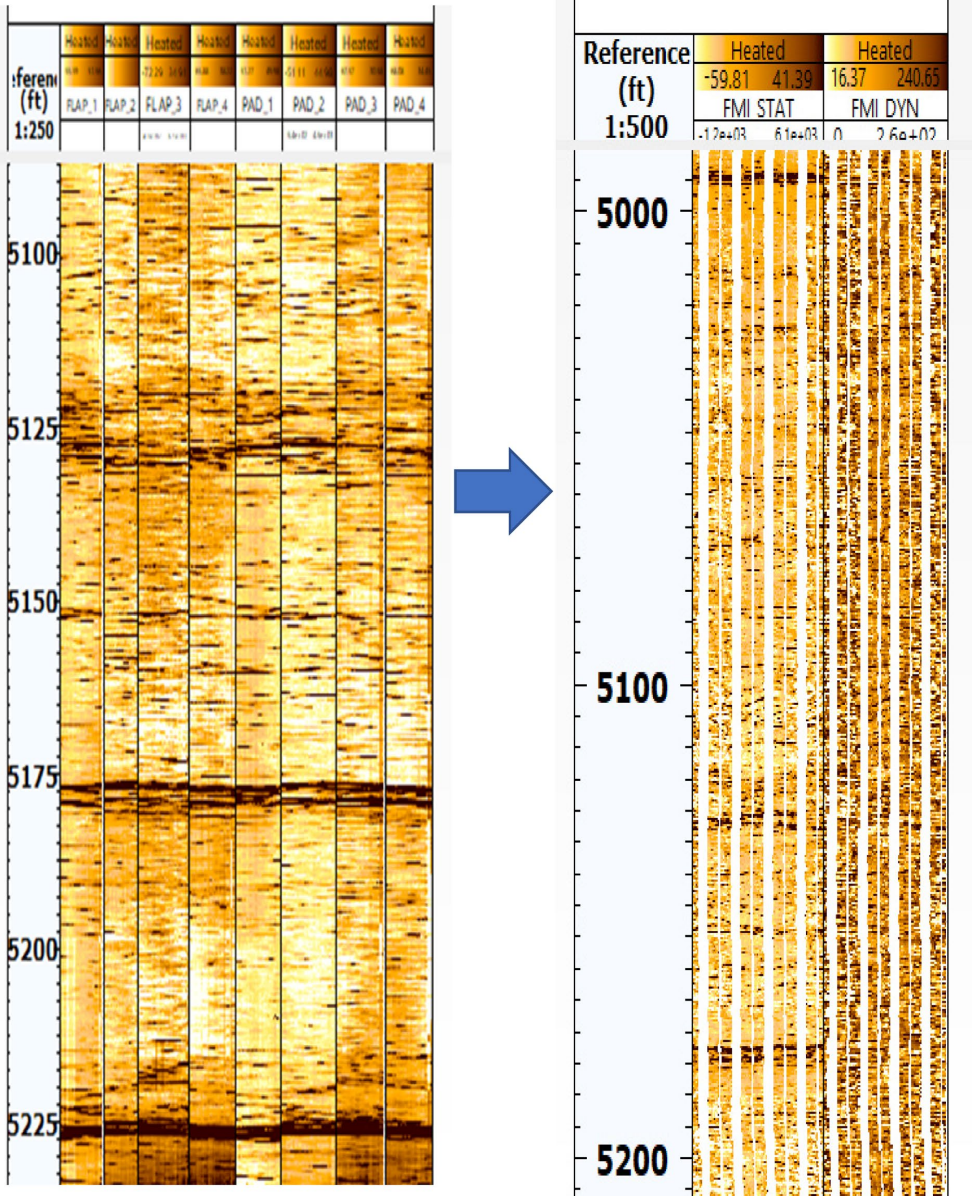
FMI data before processing (left) and processed static & dynamic images (right) for well G-21A. (generated by TechLog 2015 (Schlumberger)).

**Fig. 4:**
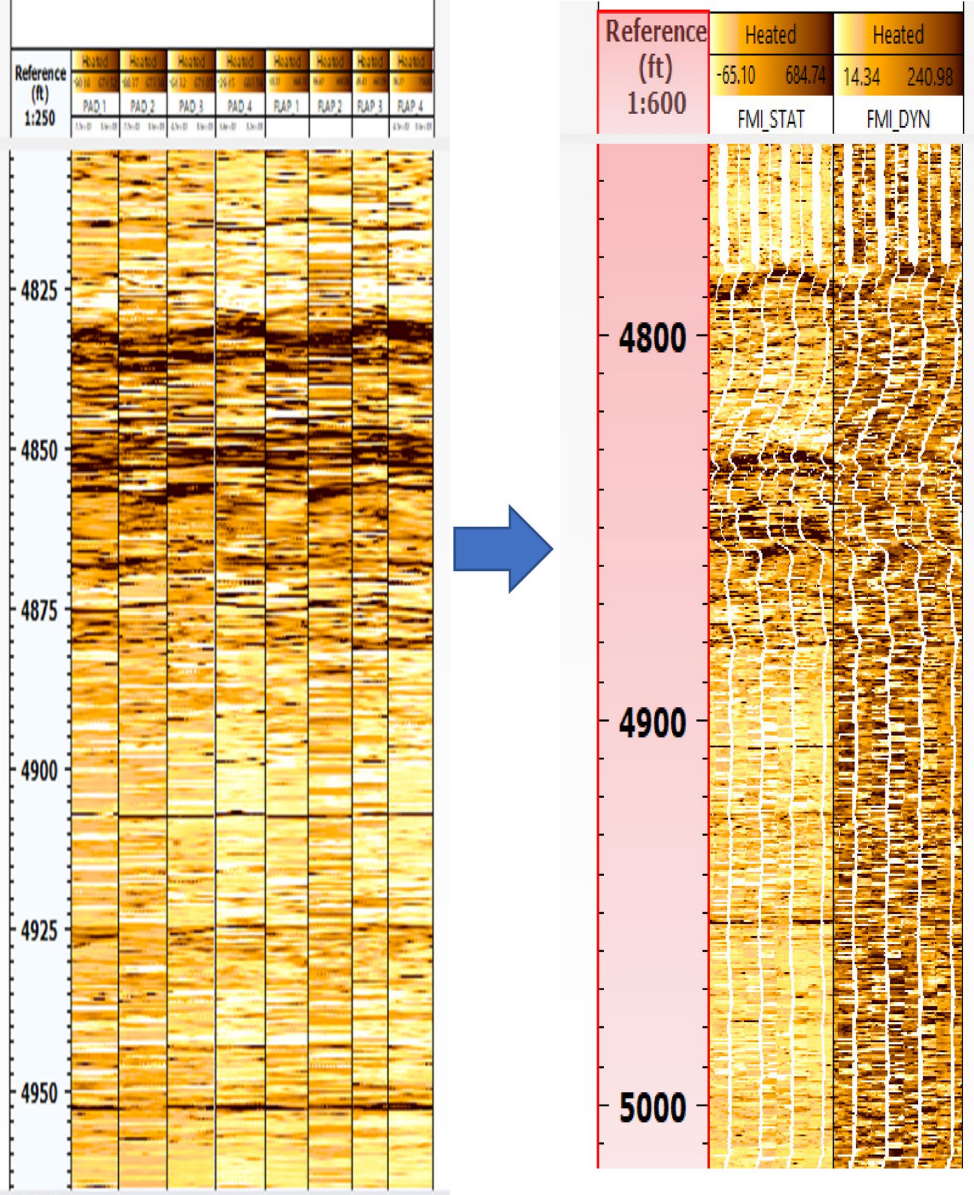
FMI data before processing (left) and processed static & dynamic images (right) for well GA-1A. (generated by TechLog 2015 (Schlumberger)).

Processing FMI data involves several critical steps to ensure accurate and reliable images of borehole walls. The first step is speed correction, which involves adjusting the vertical position of the data to account for variations in the tool’s speed. This is achieved via data from the z-axis accelerometer and an image-based correction method to align readings from the button electrodes on the pads. The second step is magnetic declination correction, which corrects for the angle between magnetic north and true north using models such as the World Magnetic Model (WMM) or the International Geomagnetic Reference Field (IGRF). Depth correction, the third step, guarantees an accurate correlation between measurements and the depth of investigation. This involves maintaining steady cable tension and recording measurements continuously as the tool string moves. The fourth step is normalization of electrode responses, which equalizes the average response of all the buttons on the tool to correct for tool and borehole effects that affect individual buttons differently. Finally, the processed data are used to generate static and dynamic normalized images, which represent conductivity changes in the borehole wall on either gray or color scales. These images provide high-resolution electrical images of the formation, aiding in the interpretation of structural characteristics, fractures, and faults^50,51^. Figures 3 and 4 show FMI data before processing and processed static and dynamic images for wells G-21A and GA-1A, respectively.

## Results and discussion

### Seismic interpretation

**Fig. 5:**
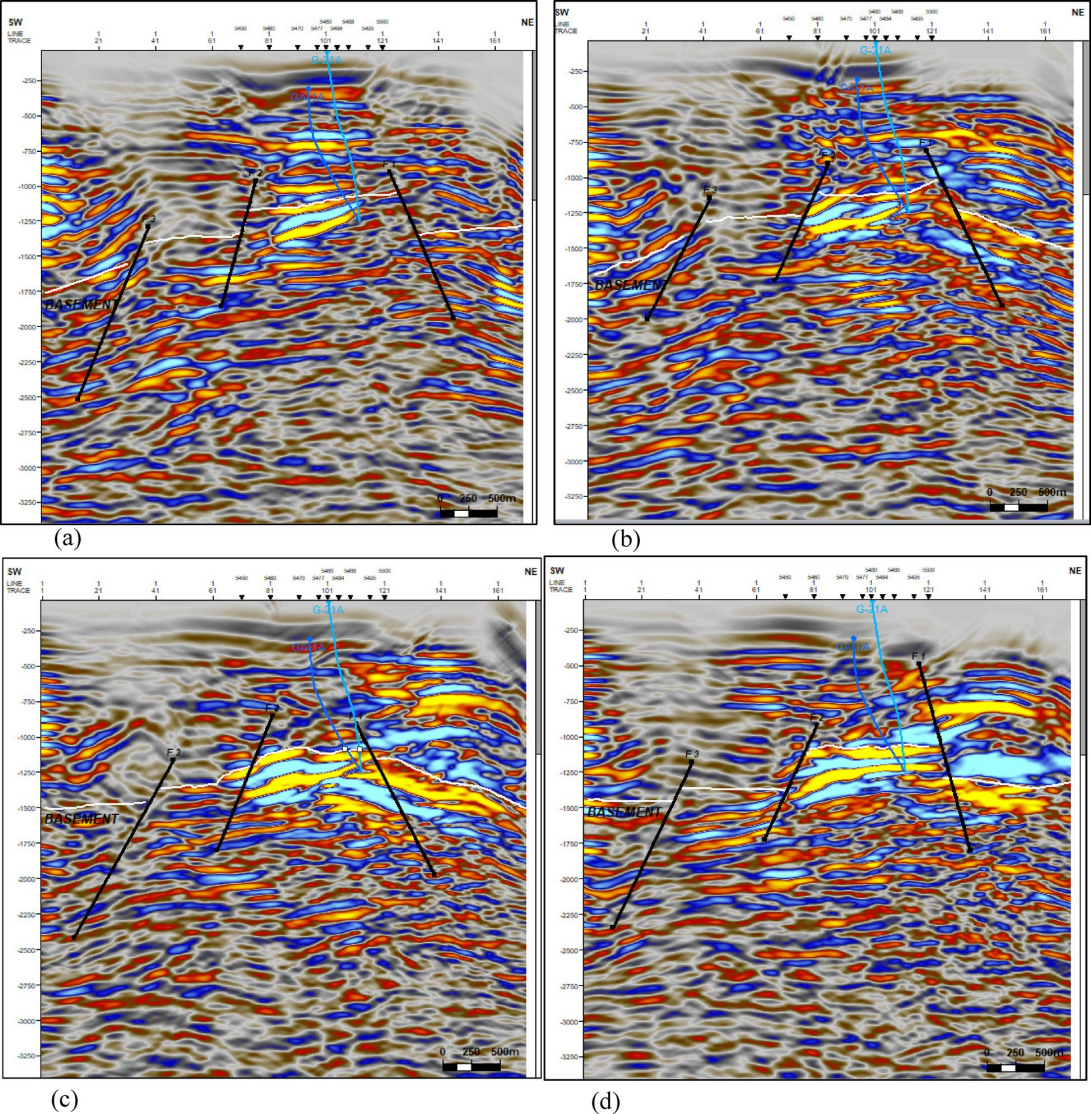
Interpreted seismic sections (**a**) 1440, (**b**) 1450, (**c**) 1460, and (**d**) 1470, showing the basement and three normal faults intersecting the study area. (generated by Petrel 2017 (Schlumberger)).

**Fig. 6:**
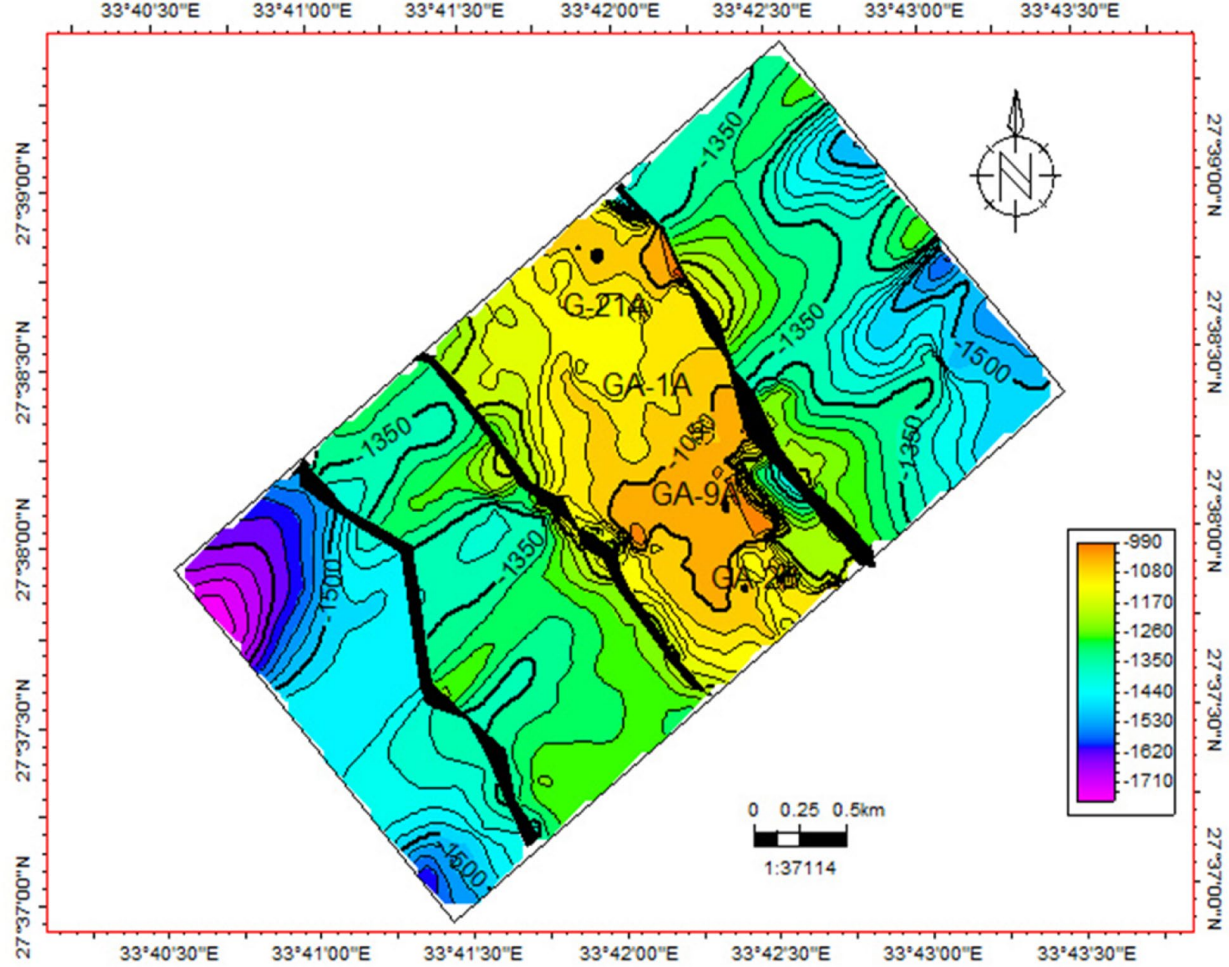
Structure contour map of the top basement rocks in the southern Geisum field. (generated by Petrel 2017 (Schlumberger)).

A checkshot survey was conducted to finalize the essential seismic-to-well connections. The basement horizon was subsequently delineated and selected for the entire region, and the faults impacting it were recognized. The research area includes three normal faults oriented from northwest to southeast. Figure 5 depicts this assembly of those faults. It depicts the configuration and characteristics of these faults, highlighting their capacity to entrap and collect hydrocarbons. This trend is crucial for continued investigations and advancements in the study domain. The wells situated at the crest structure denote the most superficial basement in the examined region. A depth–structure map was subsequently created by examining and synthesizing all accessible geological and geophysical data. The tectonic environment significantly changed the surface relief of the basement rock. Figure 6 depicts the depth‒structure contour map of the basement’s uppermost layer. In the Geisum field, the structural faults traverse from the northwest to the southeast. This fault trend forms a step-like structure that facilitates the buildup and trapping of hydrocarbons.

### Image interpretation

**Fig. 7:**
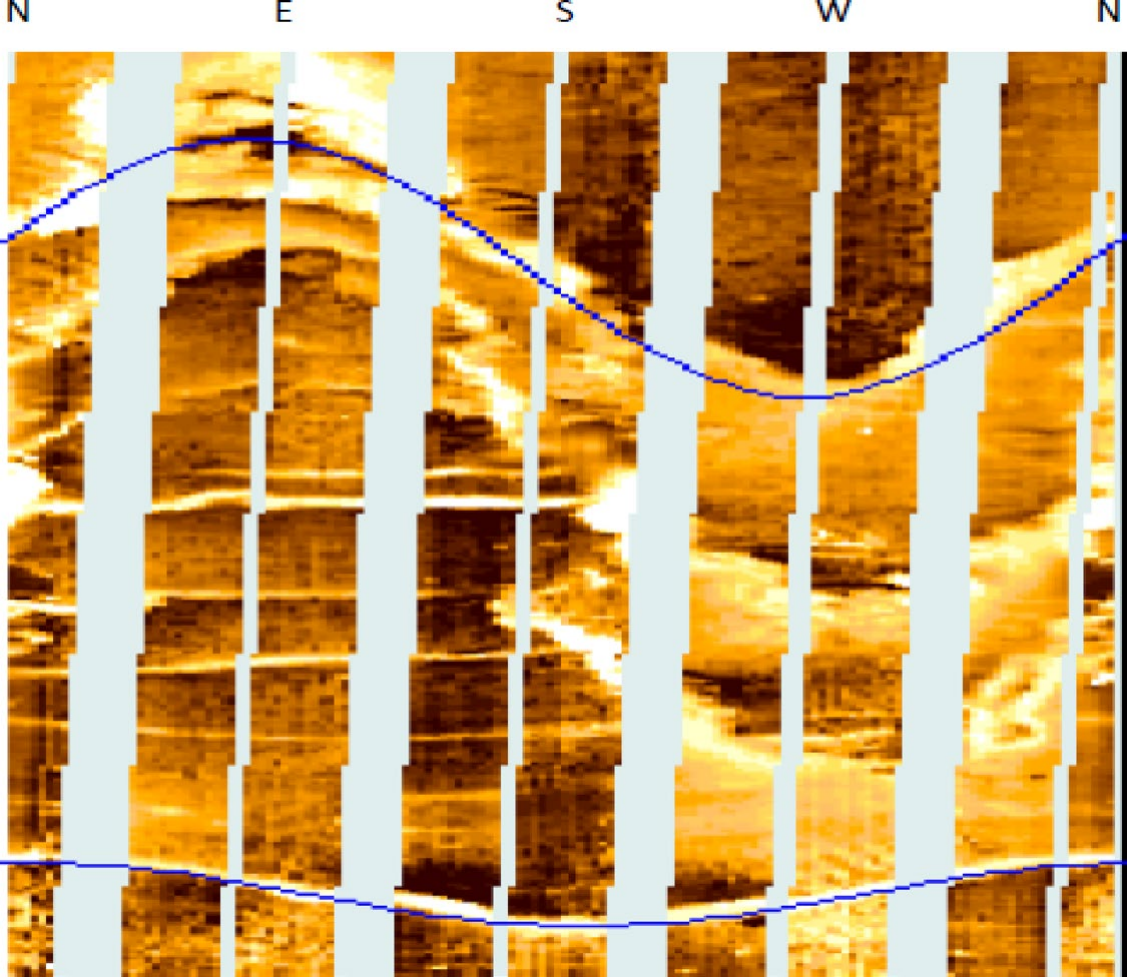
Image log showing full 360 wellbore coverage oriented from north with a sinusoidal amplitude reflecting the feature dipping angle. (generated by TechLog 2015 (Schlumberger)).

The procedure involves picking out features on the image log, depicted with a sinusoidal trace to indicate geological and structural characteristics. Each sinusoidal trace selected in the image delineates three primary categories: an angle, a depth, and an azimuth of a feature. Image log interpretation is conducted on both static and dynamic images; however, interpreting dynamic images is preferable, as color codes enhance resolution and delineate features more distinctly than static images do. The image analysis and feature grouping were conducted manually via Tech Log 2015 software. The dipping angle of the feature is directly proportional to the amplitude of the sinusoidal trace; thus, a higher dipping angle for the selected feature results in an increase in the amplitude of the sinusoidal trace (Fig. 7). We determine the dip angle for the feature by dividing the amplitude of the sinusoid by the bit size via the following equation^52^:

1 $$\text{Tan}({\alpha }) =\frac{\Delta z}{BS}$$

where α: Dipping angle (degree), Δz: sinusoidal curve amplitude (cm), BS: bit size (cm).

### Textural and structural image analysis

**Fig. 8:**
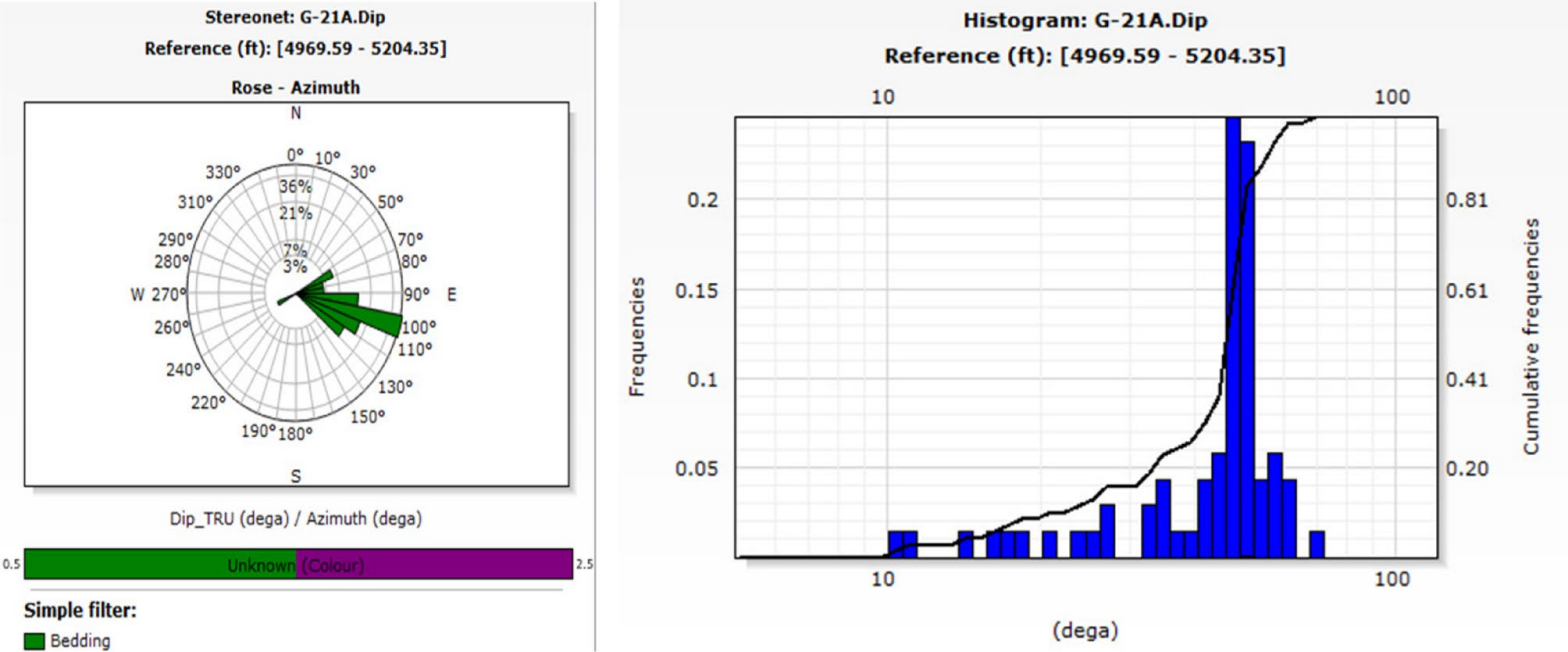
Stereo-net (left) and histogram (right) plots showing the bedding dip angles and azimuths of well G-21A. (generated by TechLog 2015 (Schlumberger)).

**Fig. 9:**
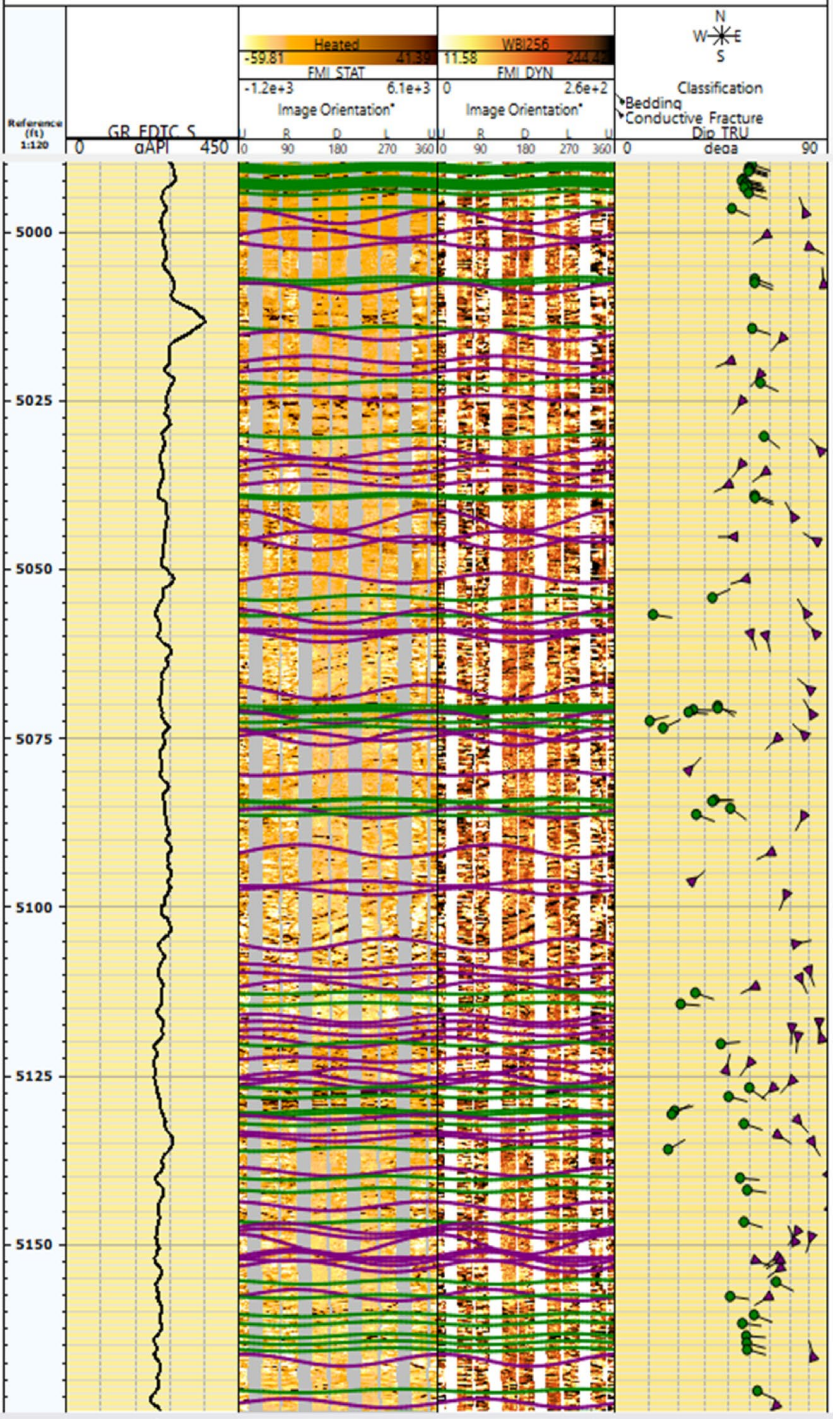
Borehole image log showing identified features (conductive fractures and bedding planes) in well G-21A. (generated by TechLog 2015 (Schlumberger)).

**Fig. 10:**
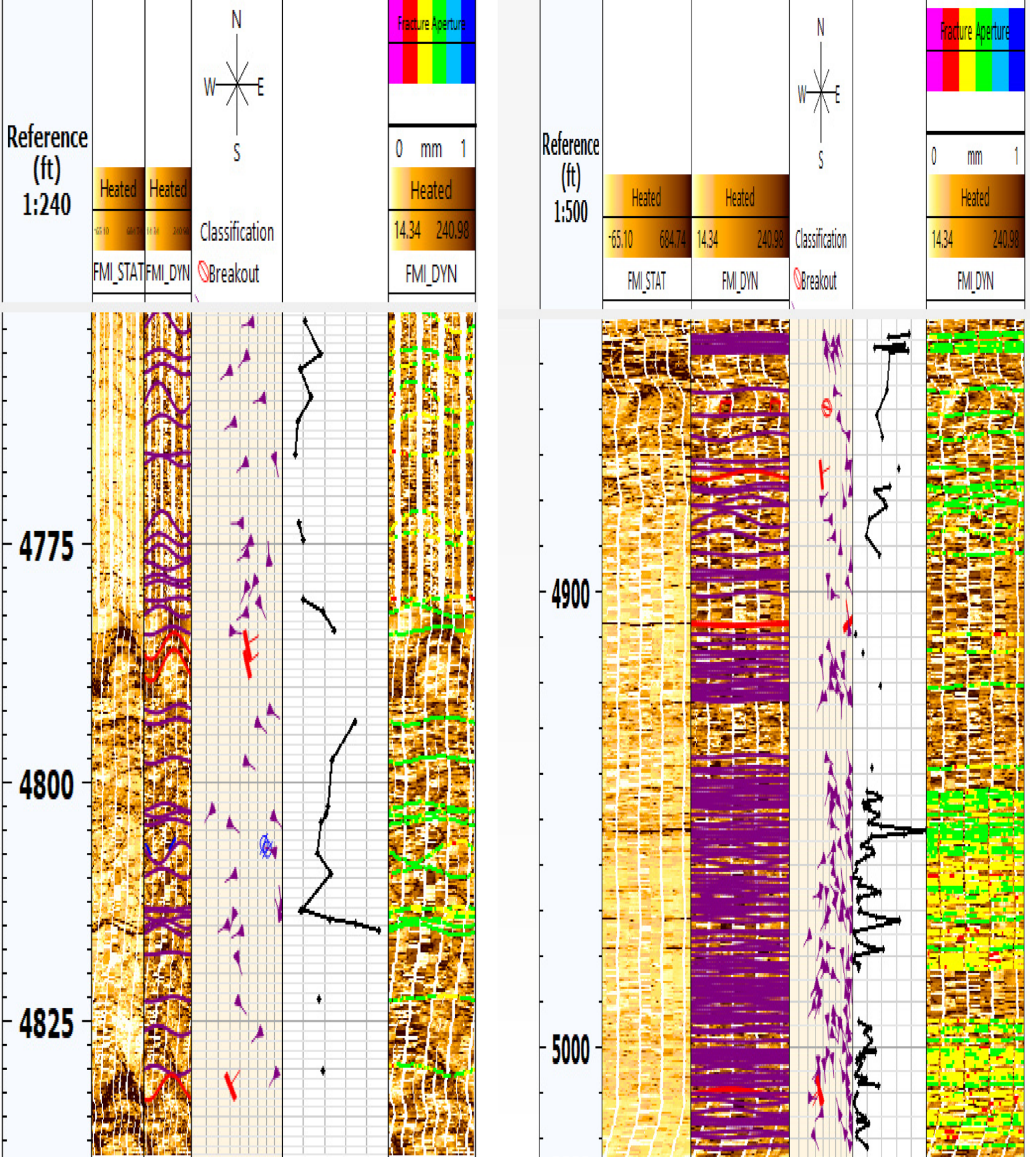
A borehole image log for well GA-1A, which focuses on geological features, primarily fractures, and their properties, such as aperture sizes. (generated by TechLog 2015 (Schlumberger)).

The process of recognizing characteristics in the image log depends on color contrast, where darker colors signify more conductive features and lighter colors indicate less conductivity. The included photos depict diverse data visualizations and analyses for two wells, G-21A and GA-1A. The selected traits included bedding planes, conductive fractures, and normal faults. The bedding plane observed in Well G-21A, indicated by the color contrast across layers that reflect diagnostic and weathering processes, influences the basement rocks in the South Geisum field. The bedding planes exhibit a severe slope toward the east. The highest frequency (~ 36%) is observed in this orientation, indicating structural alignment or bedding planes inclined toward the east. This indicates a distinct favorable orientation for sedimentary formation. Most bedding plane dip angles range from 45° to 50°, as illustrated in Fig. 8. The consistency of the dips indicates stable sedimentary deposition. The green tadpole on track 5 (Fig. 9) denotes the bedding planes selected manually. The identification of normal faults depends on the synthesis of seismic lines and image log analysis, owing to the substandard quality of seismic data resulting from attenuation by Miocene evaporite salt domes. The selection and recognition of normal faults in an image depend on many criteria. The lateral truncation of the layer in the image, the sharp shift in azimuth, and the bedding angles above and below the fault differentiate the faults. Acknowledging the red tadpole on track 4 (Fig. 10) signifies defects. The identified faults in the image are categorized into two groups: major and minor faults. The main fault strike runs from NN350°W to SS170°E and is in line with the pre-Miocene rift in the Gulf of Suez. This fault has dip angles greater than 33° and is known as the clysmic fault trend. The minor faults align N70°E–S260°W, parallel to the Gulf of Aqaba rift, with steep dip angles greater than 35°. The geological context profoundly influences the basement reservoir, where faults with a primary direction of NNW–SSE are prone to creating intricate structural traps and may function as either barriers or conduits for fluid movement, contingent upon their sealing properties. These characteristics are essential for hydrocarbon trapping mechanisms and may help define reservoir boundaries. NE-SW-oriented faults can segregate the reservoir, resulting in discrete hydrocarbon accumulations. Their existence can markedly affect fluid flow patterns in the reservoir, perhaps improving permeability if the faults are open.

The convergence of faults oriented NNW‒SSE and NE‒SW might generate intricate geological conditions, potentially resulting in heightened fracture zones and, consequently, improved porosity and permeability. Nonetheless, these zones may be vulnerable to heightened risks of fault leakage, particularly if the faults are insufficiently sealed. Such crossings necessitate meticulous assessment, as they can influence both reservoir efficacy and the structural integrity of the wellbore. Comprehending fault orientations is essential in strategizing drilling operations to prevent intersecting reactive faults, which may lead to wellbore instability or loss of zonal isolation. Directional drilling techniques must be evaluated to enhance well positioning around structural characteristics, aiming at the most productive areas while circumventing any risks linked to fault intersections. Comprehensive seismic analysis and subsurface modeling must be conducted to delineate these faults precisely. High-resolution seismic data can be used to outline fault structures and evaluate their connection and sealing capacity. This analysis is crucial for efficient reservoir management, aiding in the prediction of fluid dynamics and informing the advancement of secondary recovery methods or enhanced oil recovery procedures.

### Qualitative formation micro-image analysis

**Fig. 11:**
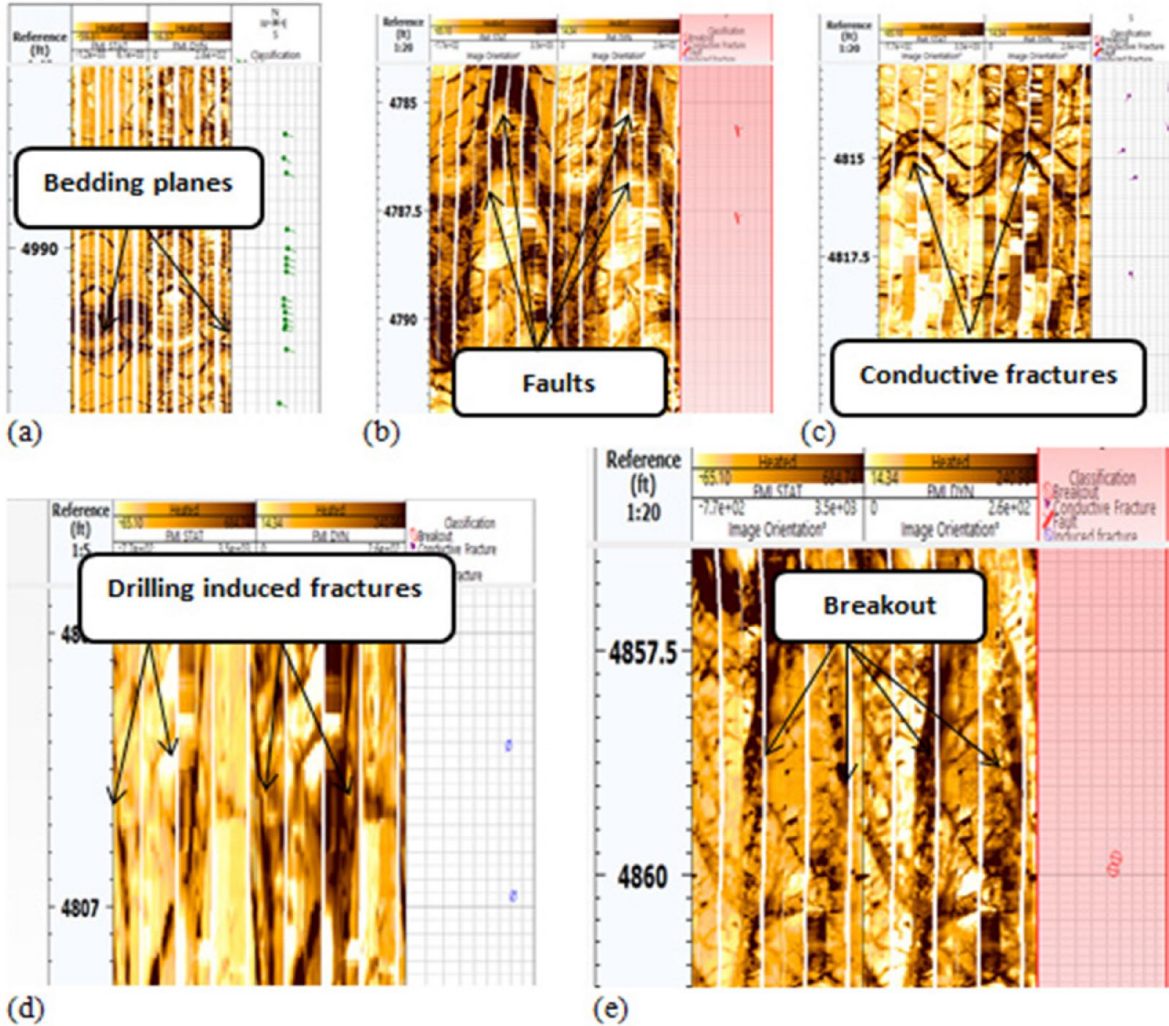
Features picked from the image log while interpreting (**a**) bedding planes, (**b**) faults, (**c**) conductive fractures, (**d**) induced fractures, and (**e**) breakouts which assist in understanding and evaluation of unconventional reservoirs. (generated by TechLog 2015 (Schlumberger)).

The identification of characteristics is conducted through visualization via resistivity contrast. Bedding planes, faults, conductive fractures, drilling-induced fractures, and breakouts are identified based on resistivity contrast and classified using a color-coding scheme, as illustrated in Fig. 11(a–e respectively). The included photos depict diverse data visualizations and analyses for two wells, G-21A and GA-1A. The pictures include stereonets, rose diagrams, histograms, and borehole image logs. Each is useful for learning about fractures and subsurface geology in its own way.

### Qualitative interpretation of conductive fractures

**Fig. 12:**
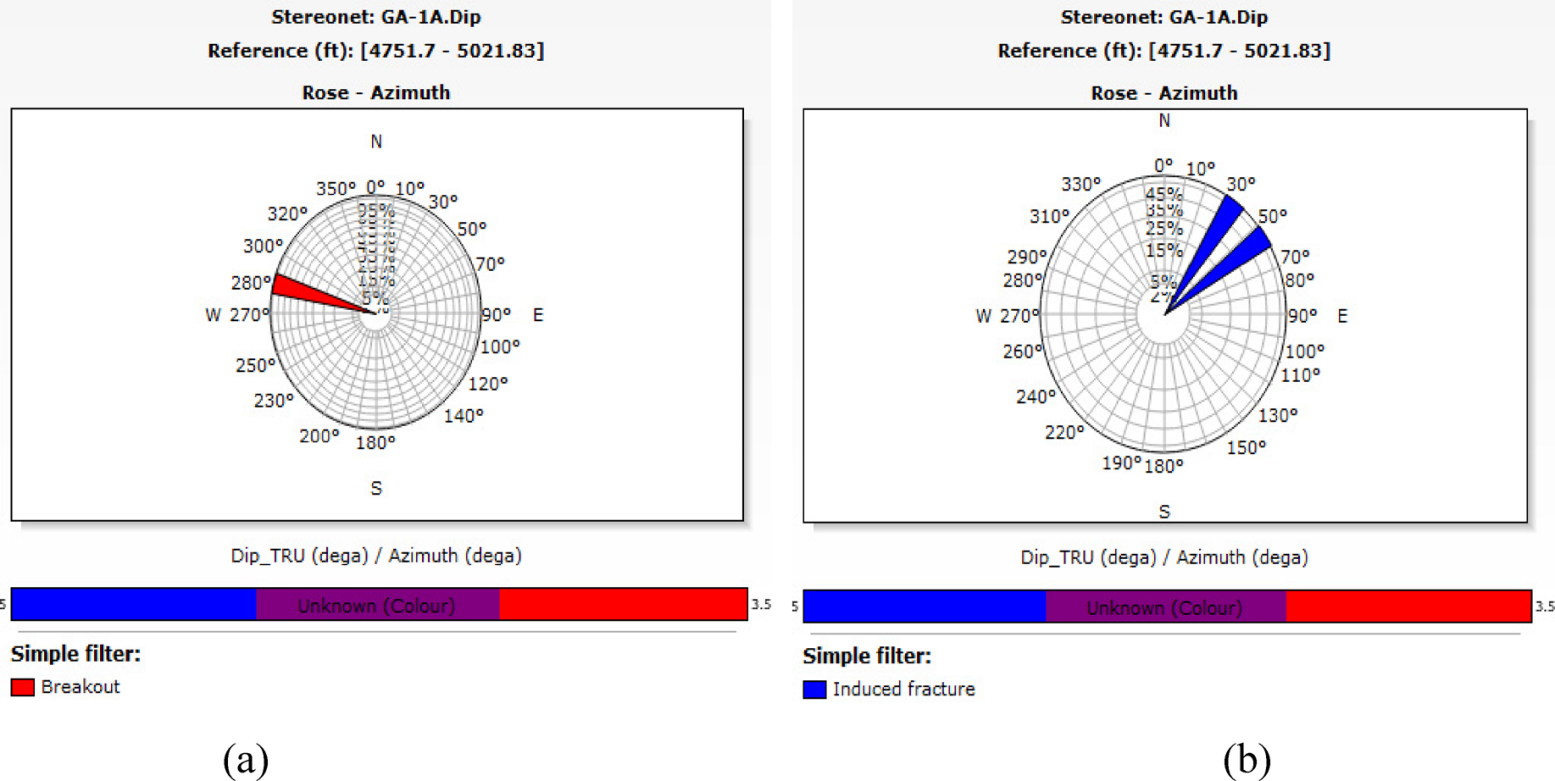
(**a**) Azimuthal orientation for breakout and (**b**) induced fracture in well GA-1A reflecting minimum and maximum horizontal stress orientation which assist in the placement of development wells trajectory.

Granitic reservoirs have been investigated by detecting and evaluating their fractures. Fractures manifest as complete sinusoidal traces on the image log when the borehole crosses the fracture at 360°. The influx of water-based mud into open fractures produces conductive fractures, which manifest as dark sinusoids on the FMI image. Fractured granite serves as an effective reservoir. The permeability and production of fractured reservoirs are strongly affected by the geological setting and the direction of natural fractures^53,54^. The defined maximum horizontal stress orientation significantly influences fluid flow behavior. The fractures align with the highest horizontal stress vector, resulting in little normal stress and consequently reducing fluid impedance to influx activity^55,56^. The breakout feature identified in the image log, oriented from northwest to southeast (Figs. 11e, 12a), aligns with the direction of minimum horizontal tension, thereby indicating the highest horizontal stress (Figs. 11d, 12b). The maximum horizontal stress, which is perpendicular to the smallest horizontal stress, is aligned from northeast to southwest. For fractured granites, two categories of fractures are recognized: (a) conductive fractures and (b) drilling-induced fractures. Analysis of the sinusoids of conductive fractures reveals that the fractures are oriented in three principal directions: (a) S250°W, (b) S140°E, and (c) N330°W. All these orientations exhibit high dip angles above 75°. Conductive fractures may affect fluid dynamics and require additional assessment. Fractures that are oriented southeast and lined up with the highest horizontal stress have a large effect on how fluids move, especially when they are connected, which makes it easier for hydrocarbons to move and build up in those directions. Fractures oriented toward the southwest may serve as conduits for fluid movement or hydrocarbon migration. High-angle fractures correspond with stress regimes, potentially indicating extensional tectonics. Minor fractures are observed in the northeast azimuth, specifically between 40° and 60°. The relative scarcity in these directions suggests less structurally controlled porosity and perhaps permeability.

### Quantitative analysis

**Fig. 13:**
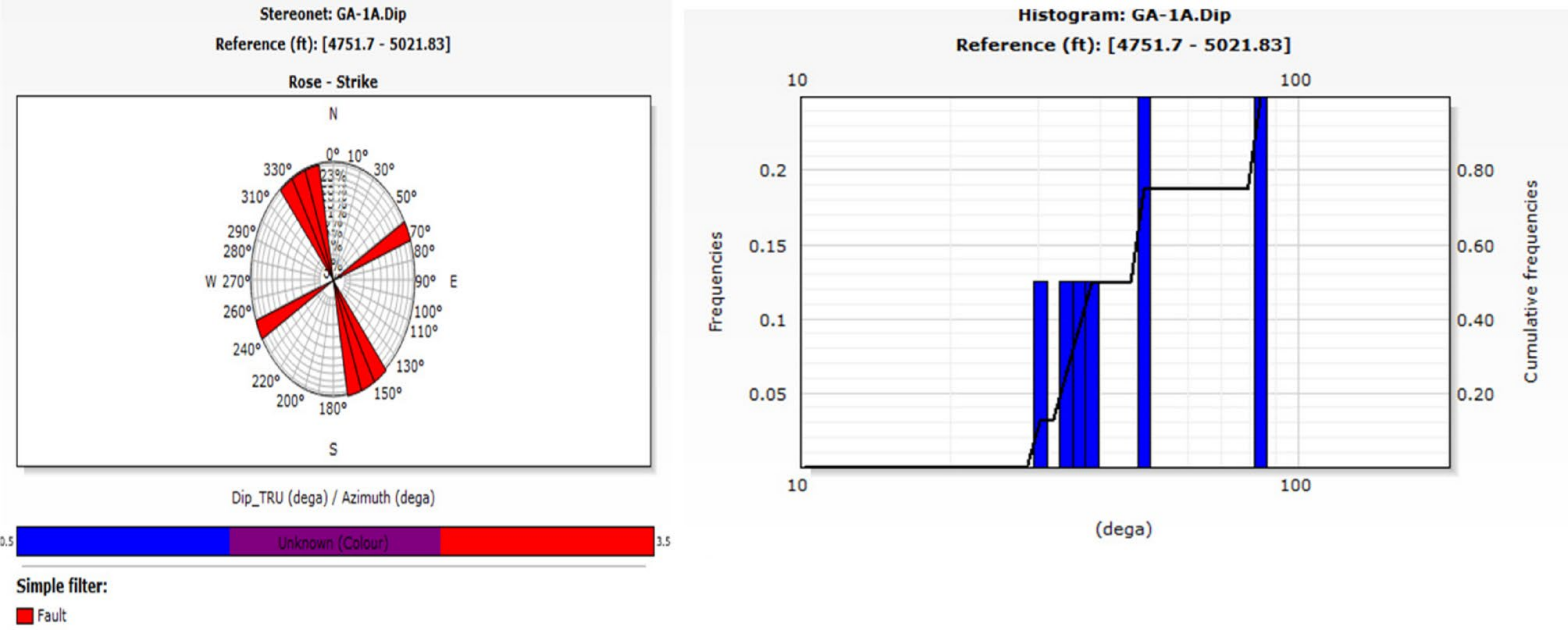
Stereo net (left) and histogram (right) showing the dip angles that exceed 30° and strikes of faults that appear orientation intersecting significantly influence fluid flow patterns within the reservoir in well GA-1A.

The bedding plane encompasses azimuths and dip angles. Figure 8 shows the stereonet diagram depicting the azimuthal orientation and dip angles for bedding plane features. The primary azimuthal directions cluster together for bedding planes between 90° and 110° (east). The highest frequency (~ 36%) is observed in this orientation, indicating structural alignment or bedding planes inclined toward the east. Most dip angles range from 45° to 50°, as illustrated in the histogram plot (Fig. 8). The consistency of the dips indicates stable sedimentary deposition. The stereonet map (Fig. 13) indicates that the principal faults are oriented NNW to SSE, with strike orientations ranging from 320° to 350° NNW and 140° to 170° SSE. The predominant fault orientation trends illustrate the influence of the Oligo-Miocene rift in the Gulf of Suez, referred to as the clysmic fault trend. Minor faults exhibit a NE‒SW trend, ranging from 60° to 70° NE and 240° to 260° SW. The subtle shifts in fault orientation indicate the influence of the Gulf of Aqaba rift. The faults exhibit significant dips, predominantly beyond 33°, as illustrated in the histogram plot (Fig. 13).

### Azimuths and dip angles for conductive fractures

**Fig. 14:**
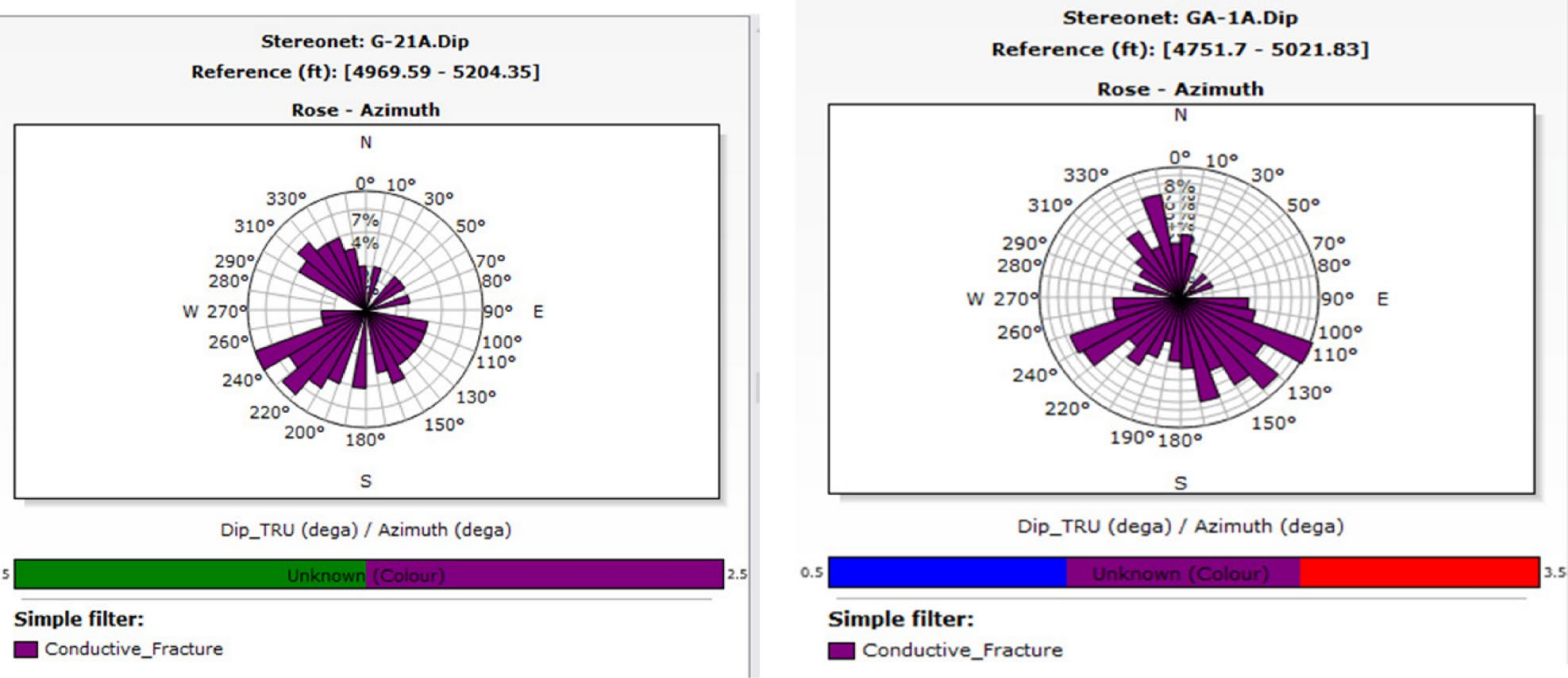
Stereo-net plots showing the conductive fracture azimuths of wells G-21A (left) and GA-1A (right).

**Fig. 15:**
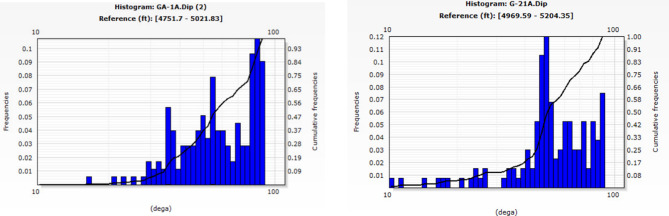
Histogram plots showing the conductive fracture dip angles of wells GA-1A (left) and G-21A (right) steeply dipping, with the majority exceeding 75°.

In well G-21A, conductive fractures predominantly occur between 220° and 250° (southwest), whereas in well GA-1A, they primarily occur between 110° and 140° (southeast). Figure 14 shows a significant prevalence of fractures in the northwest and southeast directions of the study wells. Minor fracture occurrences are noted in the northeast direction, at approximately 330°–350° in well GA-1A. The relative scarcity in these directions suggests less structurally controlled porosity and perhaps permeability. The primary fracture orientation in the east‒southeast direction may suggest the influence of regional stress fields on fracture formation. If these fractures are interconnected, they may significantly influence fluid dynamics, facilitating the migration and accumulation of hydrocarbons in those pathways. The conductive fractures exhibit significant dips, surpassing most 75, as illustrated in Fig. 15. The steep dip angle necessitated consideration of the well trajectory when new wells were positioned.

### Fracture aperture estimation

The fracture aperture measures the degree of opening of a fracture and the variables affecting its fluid conductivity and permeability. Tectonic activity, weathering processes, and chemical precipitation all influence the fracture aperture. To estimate the fracture aperture curve, it is necessary to utilize the triple-composition resistivity curve for image calibration, which can be accomplished via TechLog software. Fracture apertures $(\Delta w$) were determined via the Luthi–Souhaite equation^57^:

2 $$\Delta w = A \cdot {R}_{m}^{\left\{1-\beta \right\}}\cdot {R}_{\left\{xo\right\}}^{\left\{\beta \right\}}\cdot\Delta C$$

where A: Tool-specific calibration constant (unitless), determined from sensor specifications. Rm: Drilling mud resistivity (Ω·m), measured from fluid samples. Rxo: Flushed zone resistivity (Ω·m), derived from shallow resistivity logs. β: Empirical exponent (unitless, typically 0.75–0.85), calibrated to local lithology. ΔC: Conductivity anomaly (S/m), calculated as the difference between measured conductivity (Cactual) and background rock conductivity (Cbackground).

This equation is referred to as the Luthi–Souhaité equation^57^, named after its original inventors. The equation and associated parameters were formulated for a specific tool configuration and are hence pertinent to that tool family. The equation has been extensively utilized for calculating fracture aperture in wireline tools since its publication^57^. The equation quantifies the aperture of fractures in millimeters. An overlay of fractures is shown in the borehole image, color-coded by aperture size, with magenta denoting a low aperture and blue denoting a high aperture (Fig. 10, Tracks 5 and 6). Table 1 illustrates the fracture aperture classification and their implications for reservoir performance with key references.

**Table 1:** Fracture aperture classification and its influence on reservoir flow dynamics and performance.

Aperture width range	Category	Reservoir implications	Key references
< 0.1 mm	Microfractures	Minimal fluid flow (low permeability) Contributes to matrix storage but not drainage May act as capillary barriers if mineral-filled	^58,59^
0.1–1 mm	Mesofractures	Moderate permeability enhancement Supports drainage of low-permeability matrix Improves recovery in tight/shale reservoirs	^60,61^
1–10 mm	Macrofractures	High-permeability flow paths Dominates fluid flow (early water/gas breakthrough) Significant storage contribution	^58,62^
> 10 mm	Megafractures	Extremely high permeability Rapid aquifer invasion or gas cap expansion Minimal matrix drainage	^62,63^

### Example input values

For Well GA-1A at 4,850 ft depth:

- Rm = 0.03 Ω⋅m (measured from drilling mud sample)
- Rxo = 2.3 Ω⋅m (from shallow resistivity log)
- ΔC = 2.4 S/m (FMI-derived conductivity anomaly)
- β = 0.80 (regionally calibrated exponent)
- A = 0.25 (tool constant for Schlumberger FMI,^57,64^)

Applying the Luthi–Souhaite equation:

Δw = 0.25 × (0.03)^0.2^ × (2.3)^0.8^ × 2.4 = 0.58 mm.

### Key limitations in this study include:

- Seismic Resolution Near Salt Domes:

Velocity distortions from the salt complex introduce uncertainty in fault mapping below 4000 m depth. While pre-stack depth migration improved imaging, subtle faults may remain unresolved^65,66^.

- FMI Image Quality Assumptions:

Fracture quantification assumes optimal borehole conditions (mudcake < 0.5 cm, R_m_/R_t_ > 0.1)^67^.

- Fracture Closure Risk:

Apertures (∆w) are static measurements; overburden stress during production may reduce open fractures by 15–40% in granitic reservoirs^64^.

### Mitigation plan

To reduce these risks, we focused our analysis on intervals with good pad contact, minimal washouts, and strong image contrast. We also validated interpreted conductive fractures with resistivity separation and depth correlation to increase confidence.

### Depth zone analysis

#### Depth range: 4820 ft to 4880 ft.

Fracture aperture observations: This area has diverse fracture apertures, as demonstrated on Track 5 (Fig. 9). The fractures generally exhibit elevated aperture values in the central portion of this range, especially between 4840 and 4850 ft, when certain fractures surpass 0.7 mm in aperture.

Fault and Fracture Identification: Track 4 in Fig. 10 displays multiple conductive fractures (magenta tadpoles) and various faults (red tadpoles). Conductive cracks are more common and are distributed across the depth range, indicating zones of increased permeability.

#### Depth range: 4950 feet to 5000 feet

Observations of the fracture aperture: Fracture apertures in this range are predominantly lower, with most measurements registering below 0.4 mm. The reduced peaks in fracture aperture signify denser or less fragmented rock.

Fault and Fracture Identification: This interval, like the higher interval, encompasses both fractures and faults. Nevertheless, the frequency and distribution of these traits seem less concentrated, which may be related to the fact that the aperture size has shrunk. The examined depth intervals exhibit considerable variety in fracture aperture dimensions, with larger apertures possibly signifying enhanced reservoir quality owing to augmented fracture permeability. The existence of conductive cracks and faults in the analyzed sections indicates a complex subsurface environment with diverse consequences for fluid dynamics and reservoir management.

**Fig. 16:**
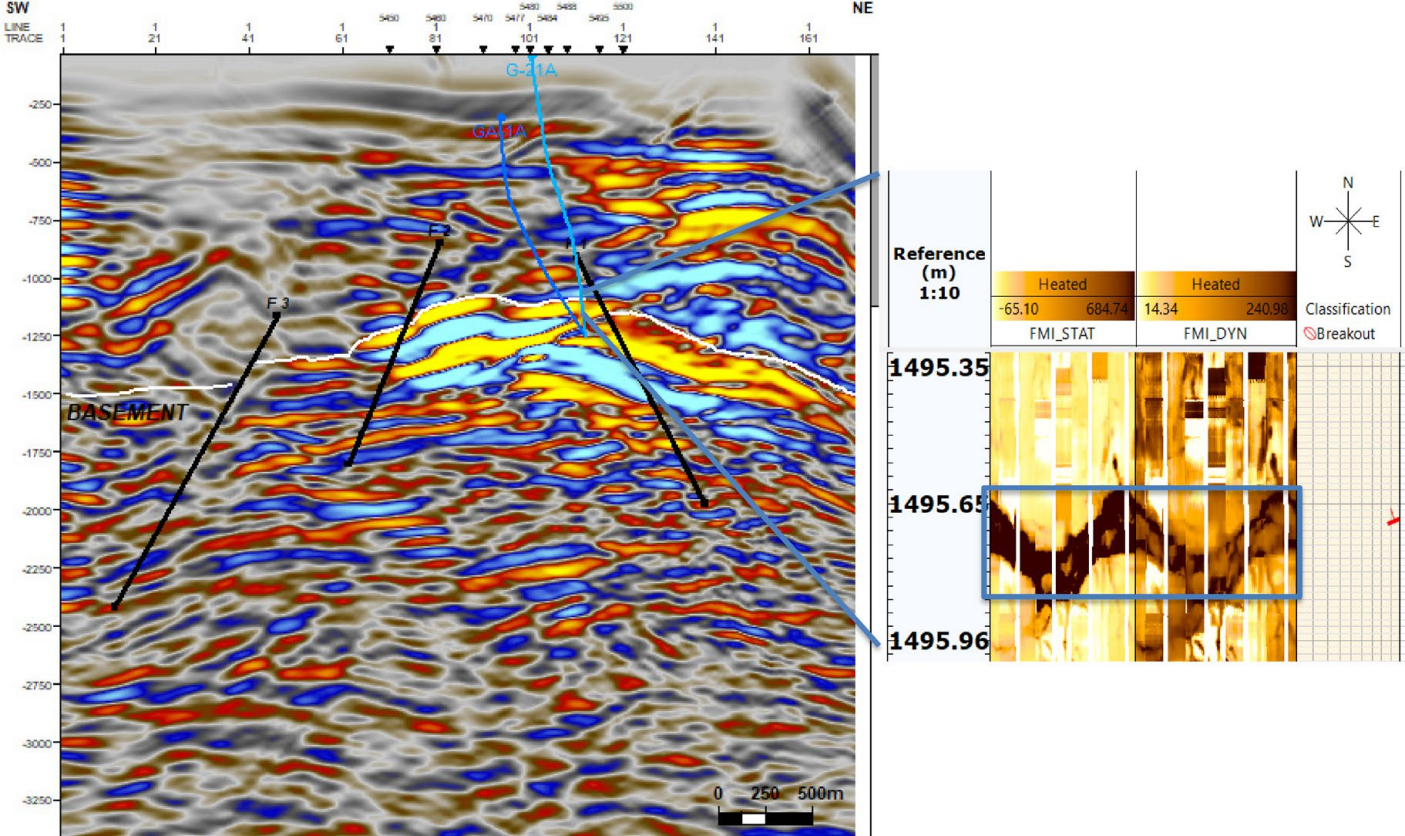
Integrated interpretation of seismic interpretation and FMI-derived fracture orientations. This figure presents a joint interpretation framework, combining seismic imaging with FMI-derived fracture orientations to enhance structural analysis. (generated by TechLog 2015 (Schlumberger) and Petrel 2017 (Schlumberger)).

The integrated interpretation presented in Fig. 16 combines seismic imaging with FMI-derived fracture orientation overlays to provide a comprehensive structural analysis of the subsurface. The seismic section delineates major fault trends and subsurface deformation patterns, offering insight into large-scale structural features. Superimposed FMI-derived fracture orientations refine this interpretation by identifying localized fracture networks, their azimuthal distribution, and intensity. This synergy between seismic and borehole-scale observations enhances the understanding of fluid migration pathways, reservoir connectivity, and potential compartmentalization within the formation. The approach is particularly effective for assessing fault-controlled reservoirs and optimizing hydrocarbon recovery strategies in structurally complex environments.

## Conclusions

In conclusion, the seismic and FMI interpretations conducted in this study provide valuable insights into the geological and structural characteristics of the research area. The delineation of the basement horizon and identification of the "clysmic fault trend" highlight the significance of these faults in hydrocarbon entrapment and collection. The depth‒structure contour map offers a comprehensive understanding of the basement surface relief and fault structures, facilitating the prediction of hydrocarbon accumulation. Image log interpretation further enhances our understanding of bedding planes, conductive fractures, and normal faults, which play crucial roles in the structural integrity and fluid dynamics of reservoirs. These findings are essential for strategizing drilling operations, optimizing reservoir management, and advancing secondary recovery methods or enhanced oil recovery procedures in the Geisum field.

- Seismic and FMI analyses delineate two dominant fault systems in the study area: major NNW-SSE faults correlating with the Gulf of Suez Oligo-Miocene rift and minor NE-SW faults aligned with the Gulf of Aqaba rift.
- These structural regimes govern hydrocarbon trapping, fluid migration, and reservoir compartmentalization. Their interplay enhances reservoir porosity/permeability but introduces fault-leakage risks, necessitating comprehensive structural analysis for optimized recovery.
- FMI resistivity contrast quantifies fracture networks, revealing: SE/SW-oriented conductive fractures parallel to maximum horizontal stress (σ < sub > H < /sub >), facilitating hydrocarbon migration, high-angle fractures indicating extensional tectonics, and minimal NE-azimuth fracturing, reducing secondary permeability.
- Quantitative fracture characterization via the Luthi–Souhaite equation shows depth-dependent permeability: Enhanced apertures (> 0.5 mm) at 4820–4880 ft depth, reduced apertures (< 0.2 mm) at 4950–5000 ft depth, and breakout analysis identifies NW-oriented minimum horizontal stress, enabling minimum horizontal stress-aligned well trajectories to stabilize boreholes and optimize drainage.
- Key Geological Insights: Seismic and FMI interpretations reveal two dominant fault systems: major NNW-SSW faults aligned with the Gulf of Suez rift, controlling hydrocarbon entrapment, and minor NE-SW faults linked to the Gulf of Aqaba rift, influencing reservoir boundaries. Their interaction creates complex traps with enhanced porosity but leakage risks.
- Innovative Fracture Characterization: We pioneer quantitative fracture analysis in Geisum’s basement reservoir using FMI resistivity contrast and the Luthi–Souhaite equation. Conductive fractures, oriented SE/SW align with maximum horizontal stress (σH), facilitating fluid flow. Breakout analysis identifies σh orientation (NW), enabling optimized well placement.
- Depth-Specific Reservoir Properties: High-permeability zones (4820–4880 ft) correlate with wider fracture apertures (> 0.5 mm), while denser intervals (4950–5000 ft) show narrower fractures (< 0.2 mm). This depth-dependent variability directly informs targeted drilling and EOR strategies.
- Advantages & Disadvantages

Advantages:

- Integrated seismic/FMI analysis reduces interpretation uncertainty.
- Dual fault mapping clarifies trap-leakage dynamics.
- σh-oriented well designs enhance drilling safety.

Disadvantages:

- Limited well coverage may overlook fracture heterogeneity.
- Fracture aperture estimates assume uniform mineral fill.
- FMI cannot distinguish open vs. mineralized fractures without core calibration.

- Future research directions
  - Fracture Validation: Core-flood experiments to calibrate FMI-derived aperture models.
  - Dynamic Monitoring: 4D seismic tracking of fluid migration along fault-fracture networks.
  - Geomechanical Modeling: Simulate stress evolution during production to predict seal integrity.
  - Machine Learning Applications: Fracture prediction in unexplored blocks using seismic attributes and FMI data.

## Data Availability

The current study’s datasets are not publicly available, but the corresponding author can provide them upon request.
